# Association of FTO Polymorphisms with Obesity and Metabolic Parameters in Han Chinese Adolescents

**DOI:** 10.1371/journal.pone.0098984

**Published:** 2014-06-09

**Authors:** Junqing Wu, Jianhua Xu, Zhaofeng Zhang, Jingcao Ren, Yuyan Li, Jian Wang, Yunlei Cao, Fen Rong, Rui Zhao, Xianliang Huang, Jing Du

**Affiliations:** 1 WHO Collaborating Center on Human Research, Shanghai Institute of Planned Parenthood Research, Shanghai, China; 2 NPFPC Key Laboratory of Contraceptives and Devices, Shanghai Institute of Planned Parenthood Research, Shanghai, China; 3 Institute of Reproduction & Development, Shanghai Medical College, Fudan University, Shanghai, China; 4 School of Public Health, Xinxiang Medical University, Xinxiang City, Henan, China; Mathematical Institute, Hungary

## Abstract

**Background:**

Previous studies have suggested that fat mass-and obesity-associated (FTO) gene is associated with body mass index (BMI) and the risk of obesity. This study aims to assess the association of five FTO polymorphisms (rs9939609, rs8050136, rs1558902, rs3751812 and rs6499640) with obesity and relative parameters in Han Chinese adolescents.

**Methods:**

We examined a total of 401 adolescents, 223 normal weights (58.7% boys, 41.3% girls), 178 overweight (60.1% boys, 39.9% girls), aging from 14 to 18-years-old, recruited randomly from public schools in the central region of Wuxi, a southern city of China. DNA samples were genotyped for the five polymorphisms by Sequenom Plex MassARRAY. Association of the FTO polymorphisms with BMI, serum fasting plasm glucose (FPG), fasting insulin (FIns), triglyceride (TG) and cholesterol (TC) were investigated.

**Results:**

1) Serum FPG, FIns, TG and TC were statistically significant higher than that in normal control group. 2) We found that BMI was higher in the rs9939609 TA+AA, rs8050136 AC+AA, rs1558902 TA+AA and rs3751812 GT+TT genotypes than in wild TT genotypes (rs9939609: *P* = 0.038; rs1558902: *P* = 0.038;), CC genotypes(rs8050136: *P* = 0.024) and GG genotypes (rs3751812: *P* = 0.024), which were not significant on adjusting for multiple testing. 3) In case-control studies, five polymorphisms were not significantly associated with overweight (*p*>0.05), haplotype analyses showed non-haplotype is significantly associated with a higher risk of being overweight (*p*>0.05). 4) There existed no significant statistical difference about FPG, FIns, TG and TC in genotype model for any SNP.

**Conclusions:**

Our study has conducted a genetic association study of the FTO polymorphisms with BMI, serum fasting plasm glucose (FPG), fasting insulin (FIns), triglyceride (TG) and cholesterol (TC). Our study found BMI of subjects with A allele of FTO rs9939609 is higher than that with T allele. Further studies on other polymorphisms from FTO and increasing the sample size are needed.

## Introduction

Childhood obesity is an increasing public health issue worldwide including the developing countries like China [1]. The prevalence rates of overweight in 1985 from the metropolis areas were between 1% and 2%. The rates of obesity were only 0.2% and 0.1%, respectively for boys and girls. Around 1995, in the most developed metropolis, the prevalence of overweight was two to three folds more than that of 10 years earlier. The prevalence of obesity were from 6% to 8% for boys and from 4% to 6% for girls, respectively. Since 2000, the prevalence rates of obesity plus overweight had reached 25.4%, 25.5%, 17.0% and 14.3% for boys aged 7–9 years and 10–12 years, and girls aged 7–9 years and 10–12 years, respectively [2]. The report from Guangzhou (one of the most urbanized areas in China) showed that the total prevalence of adolescent overweight and obesity increased from 8.1% and 3.1% in 2007 to 10.0% and 4.2% in 2011, respectively. Although the prevalence of adolescent overweight and obesity in Guangzhou in 2011 is still lower than the average values of Chinese large coastal cities, a significant increase was found in their prevalence from 2007 to 2011 [3]. Overweight and obesity are major health issues associated with the risk factors of hypertension, type II diabetes and cardiovascular diseases [4], [5]. In addition to environmental factors, genetic factors also clearly contribute to obesity-related phenotypes, with heritability estimates ranging from over 50% to 60% for body mass index (BMI) [6], [7].

Until now, at least 52 loci associated with obesity risk and obesity-related traits have been identified through GWAS [8]. Since 2007, an association between FTO single nucleotide polymorphisms (SNPs) and body mass index (BMI) and the risk of obesity had been identified in multiple populations, including adolescents and children. So far, FTO is considered to be the first locus unequivocally associated with adiposity. Several SNPs have been described in this gene, especially a T-to-A change in the first intron (rs9939609) is the most widely investigated and is consistently associated with obesity-related phenotypes, mainly the body mass index (BMI). Each rs9939609-A allele in this gene increases body weight by 1.5 kg in adult, with similar effects observed in children and adolescents [9].

Multitudinous studies have associated FTO polymorphisms with obesity in different European populations [10]. However, the contribution of the FTO common variants to obesity is controversial in Han Chinese, some studies showed rs9939609 was statistically associated with BMI [11], but other results reported FTO gene is not statistically associated with obesity [12]. Thus, the aim of this study was to evaluate the association between five FTO SNPs, including rs9939609, rs1558902, rs8050136, rs3751812 and rs6499640 with the susceptibility to obesity in Han Chinese adolescents.

## Materials and Methods

### Study Subjects

We set up a case control study from the cohort. For each case, one control subject with birth weight 2500–4000 g, matched frequently by year of birth, sex of infant, type of institute at birth (township, regional central and tertiary centre) were chosen. The parents who agreed to participate after full explanation of the purposes and procedures of the study were asked to sign consent. This study was approved by the Ethics Committee of Shanghai Institute of Planned Parenthood Research/WHO Collaborating Center on Human Research. Han adolescents aging 14 to 18 years old were randomly selected from eight public schools of three districts in Wuxi of Jiangsu Province (southern city of China). A total of 401 adolescents comprising of 238 boys and 163 girls were recruited, and were classified using age and sex specific BMI cut-offs provided by Working Group of Obesity in China (WGOC) [13]. From the 401 analyzed adolescents, two BMI groups were formed: 178 subjects (60.1% boys, 39.9% girls) were classified as overweight group and 223 subjects (58.7% boys, 41.3% girls) as normal control group.

### Anthropometric Measurements

All investigators were specially trained to control the quality of the measurement. Height (cm) and weight (kg) were taken with participants dressed in lightweight clothing without shoes. Waist circumference (cm) was measured midway between the lowest rib and the iliac crest, to the nearest 0.1 cm after inhalation and exhalation. Hip circumference (cm) was measured at the point over the buttocks yielding the maximum circumference. The BMI was calculated with the weight in kilograms divided by the square of height in meters (kg/m^2^).

Venous blood samples were drawn after at least 10 hours of overnight fasting. Serum and plasma samples were frozen and stored at −70°C until the tests were performed. Plasma glucose (FPG, mg/dl), total cholesterol (TC, mmol/l) and triglycerides (TG, mmol/l) were assessed by standard laboratory methods using HITACHI7180 biochemistry automatic analyzer (HITACHI, Japan). Insulin (µU/ml) was measured by an enzyme-linked immunoassay kit (CRYSTAL CHEM, USA).

### Selection of SNPs and Genotyping

Samples were analyzed for five variants within intron of the FTO gene: rs9939609 (A/T), rs1558902 (A/T) and rs8050136 (A/C) were reported to be associated with BMI,whereas rs6499640 (A/G) and rs3751812 (G/T) were associated with weight [9], [14], [15].

Genomic DNA was isolated from blood leukocytes with QIA-amp DNA Blood Kit (QIAGEN, Hilden, Germany) according to the manufacturer's instructions. Genotyping was performed without the knowledge of the clinical status of the subjects.

SNP genotyping was performed using the Sequenom iPlex MassARRAY platform according to manufacturer's instructions (Sequenom, San Diego, CA). A 90% sample quality control (QC) rate and 90% SNP genotyping success rate were imposed on the analysis.

### Statistical Analysis

Genotype and allele frequencies for the case and control groups were compared using the χ^2^ test which was performed using SPSS (version 19.0). Linkage disequilibrium statistics were computed using D' and r^2^ tested with Haploview. The odds ratio (OR) and their 95% confidence intervals (95% CI) were calculated through the estimation of the effects of alleles. The Hardy-Weinberg equilibrium test was performed using STATA (version10.0). Haplotype frequencies were estimated using SHEsis (http://analysis.bio-x.cn/myAnalysis.php). P values were adjusted by Bonferroni method. A value of P<0.05 was considered statistically significant.

## Results

The analyzed adolescents were divided into two groups according to the definition of BMI specified by WGOC [9]. From a total of 401 adolescents measured for anthropometric traits, genotyping was performed in 178 subjects classified as overweight group and 223 as normal control group. Genotype frequencies for the total sampled population were in accordance with Hardy-Weinberg equilibrium.

The characteristics of the adolescents were shown in Table 1.The levels of FPG, Fins, TG and TC were significantly higher in overweight group than that in normal control group.

**Table 1:** General characteristic of the sampled adolescents by phenotype distribution.

Characteristics	Phenotype	distribution
	Normal	Overweight
N	223	178
Gender(M/F)	131/92	107/71
Age(years)	16.3±1.5	16.2±1.7
Height(cm)	169±9.6	168.5±8.5
Weight(kg)	56±4.9	70.9±9.9
BMI(kg/m^2^)	19.6±1.5	25.1±1.6*
Waist circumference(cm)	71.12±5.01	78.98±5.42
Hip circumference(cm)	88.43±7.62	93.23±8.11
TC(mg/L)	4.13±0.81	4.43±0.89*
TG(mmol/L)	1.11±0.53	1.40±0.61*
FPG(mmol/L)	4.79±0.92	5.04±1.12*
FIns(U/L)	8.45±5.29	10.38±8.23*

Notes M: male; F: female; * P<0.05.

We performed association analysis using BMI case-control groups. The data for genotypes and allele frequencies are shown in Table 2. During association analysis under allelic model and genotype model, we detected no significant association when comparing overweight *vs*. normal-weight groups (*p*≥0.05) (Table 2).

**Table 2:** Allele and genotype frequencies of FTO genetic variants in overweight (n = 178) and controls (n = 223).

	ALLELE		*P*	OR (95%CI)	GENOTYPE			*P*	Hardy-Weinberg equilibrium test
**rs9939609**	A	T			A/A	A/T	T/T		
case	48(0.136)	304(0.864)	0.07	1.5	3(0.017)	42(0.239)	131(0.744)	0.16	0.86
control	42(0.095)	398(0.905)		(0.96∼2.32)	1(0.005)	40(0.182)	179(0.814)		0.43
**rs1558902**	A	T			A/A	A/T	T/T		
case	48(0.136)	304(0.864)	0.09	1.46	3(0.017)	42(0.239)	131(0.744)	0.19	0.86
control	43(0.098)	397(0.902)		(0.94∼2.26)	1(0.005)	41(0.186)	178(0.809)		0.4
**rs8050136**	A	C			A/A	A/C	C/C		
case	49(0.138)	305(0.862)	0.06	1.52	3(0.017)	43(0.243)	131(0.740)	0.14	0.81
control	42(0.095)	398(0.905)		(0.98∼2.36)	1(0.005)	40(0.182)	179(0.814)		0.43
**rs3751812**	T	G			T/T	G/T	G/G		
case	49(0.138)	305(0.862)	0.06	0.66	3(0.017)	43(0.243)	131(0.740)	0.15	0.81
control	42(0.096)	396(0.904)		(0.43∼1.02)	1(0.005)	40(0.183)	178(0.813)		0.43
**rs6499640**	A	G			A/A	A/G	G/G		
case	56(0.159)	296(0.841)	0.98	1.01	6(0.034)	44(0.250)	126(0.716)	0.99	0.38
control	70(0.158)	372(0.842)		(0.69∼1.47)	8(0.036)	54(0.244)	159(0.719)		0.22

To calculate the extent of linkage disequilibrium (LD) in pairwise combinations of the 5 SNPs, we calculated D' and r ^2^, the normalized LD statistic for all possible pairs of SNPs. The pairwise D' values are shown in Table 3. Strong LD among the four SNPs (rs9939609, rs8050136, rs1558902 and rs3751812) was observed (D'>0.9).

**Table 3:** Pairwise linkage disequilibrium.

D':	rs6499640	rs9939609	rs1558902	rs8050136
rs3751812	0.245	0.975	0.975	0.975
rs6499640	-	0.261	0.254	0.261
rs9939609	-	-	1.000	1.000
rs1558902	-	-	-	1.000

Haplotype analysis associating the four studied FTO SNPs (rs9939609, rs8050136, rs1558902 and rs3751812), revealed all the five possible haplotypes, being the most commons GTTC (89%) and TAAA (13%) (Table 4). Three haplotypes had an estimated frequency below 3% (GAAA, GTAC and TTTC). The results showed no statistical association between overweight group and control group (*p* = 0.055);

**Table 4:** Haplotype frequencies of FTO genetic variants in overweight (n = 178) and controls (n = 223).

Haplotype	case	control	*P*	OR	95% CI
GTTC	304(0.0.864)	390.99(0.897)	0.055	0.648	0.42∼1.01
TAAA	48(0.136)	39.99(0.092)	0.055	1.544	0.99∼2.41

We analyzed anthropometric traits among different genotypes of FTO SNPs (Table 5), and found that BMI was higher in the rs9939609 TA+AA, rs8050136 AC+AA, rs1558902 TA+AA and rs3751812 GT+TT genotypes than in the wild TT genotypes (rs9939609:*P* = 0.038; rs1558902: *P* = 0.038;), CC genotypes(rs8050136: *P* = 0.024) and GG genotypes (rs3751812: *P* = 0.024), which were found to be not associated when adjusted for multiple test. No significant differences in BMI between the rs6499640 AG+AA and GG genotypes were observed. However, FPG, FIns, TG and TC showed no significant differences in genotype model for any SNP.

**Table 5:** The association of FTO gene SNP with obesity related parameters.

Parameters	rs9939609	genotype	*p*	rs8050136	genotype	*p*	rs3751812	genotype	*p*	rs1558902	genotype	*p*	rs6499640	genotype	*p*
	TT(310)	TA(82)+ AA(4)		CC(310)	AC(83)+ AA(4)		GG(309)	GT(83)+ TT(4)		TT(310)	TA(82)+ AA(4)		GG(285)	AG(98)+ AA(14)	
Gender (M/F)	186/124	48/38		186/124	47/40		184/125	49/38		186/124	48/38		171/114	64/48	
BMI (kg/m^2^)	21.87±3.68	22.58±3.89	0.038*	21.86±3.56	22.59±3.43	0.024*	20.44±3.52	22.58±3.49	0.024*	21.87±3.68	22.58±3.89	0.038*	21.95±3.86	22.14±3.79	0.28
TC(mg/L)	4.27±0.71	4.25±0.76	0.46	4.27±0.69	4.25±0.73	0.43	4.17±056	4.27±0.68	0.48	4.27±0.71	4.25±0.76	0.46	4.29±0.70	4.19±0.74	0.15
TG (mmol/L)	1.28±0.62	1.10±0.61	0.08	1.27±0.60	1.11±0.59	0.09	1.20±0.46	1.23±0.56	0.17	1.28±0.62	1.10±0.61	0.08	1.25±0.63	1.21±0.62	0.36
FPG (mmol/L)	4.92±0.69	4.87±0.54	0.32	4.93±0.66	4.86±0.49	0.28	4.80±0.61	4.86±0.55	0.29	4.95±0.68	4.79±0.53	0.06	4.92±0.69	4.87±0.54	0.32
Fins (U/L)	9.29±4.62	10.64±5.23	0.12	9.3±4.35	10.70±5.13	0.11	8.99±4.23	10.65±4.89	0.13	9.29±4.62	10.64±5.23	0.12	9.83±4.61	8.86±5.25	0.18

Notes M: male; F: female;

After adjusting for multiple test, *P*>0.05.

## Discussion

Due to near complete linkage disequilibrium, results follow the same pattern for four SNPs (rs9939609, rs8050136, rs1558902 and rs3751812). As the *P* values were more often found significant for the rs9939609, we will focus here on the results concerning the rs9939609 polymorphism.

Frayling et al. firstly reported that rs9939609 in the first intron of FTO showed a significant association with obesity-related traits in adults and children of European descent [9]. Since then, multitudinous studies have confirmed the association between FTO and BMI in populations of Caucasian children and adults [16], [17], but negative results were found in some studies regarding populations of Oceanic [18], African [19] and Asian ancestries [12]. In the Chinese population, Chang et al reported that rs9939609 A allele was strongly associated with obesity and BMI [11]. However, the populations of Beijing and Shanghai adults, it has been observed that FTO gene is not associated with obesity [12]. The contradictory results among different ethnic populations are likely the result of varying degrees of linkage disequilibrium between SNPs, which suggests that the underlying causative variant is being tagged differently by FTO in these populations [16], [19], [20] or that there are different gene-environment interactions.

Other studies have suggested that FTO SNPs are associated with metabolic traits (FPG, FIns, TG and TC) that are mediated through BMI. Thus, the genetic information may be useful to identify high-risk children who may need early interventions such as lifestyle modifications and individualized management strategies, in order to reduce the incidence of obesity related diseases.

The mechanisms of how BMI associated SNPs influence obesity are unclear. FTO proteins are highly expressed in hypothalamus, mainly in the arcuate nucleus, which regulates the energy balance [21]. Many studies have indicated that FTO variants influence energy-dense food intake rather than regulation of energy expenditure[22]. The study showed that reduced fat mass in FTO-deficient mice was not due to reduced food intake, but higher levels of energy expenditure. The increase in energy expenditure was unrelated to the levels of physical activity but was potentially mediated by increased sympathetic nervous system (SNS) activity [23]. Gao et al. generated a nervous system FTO specific knockout mice, and obtained a similar phenotype [24]. They concluded that FTO functions in the central nervous system to regulate postnatal growth [23], [24]. Mice carrying extra copies of FTO have a dose dependent increase in body weight due to increased adipose tissue mass and adipocyte size. These overexpression mice are hyperphagic and when fed a high fat diet have an increased glucose tolerance and increased fasting insulin [25]. These mouse models suggest that FTO plays a role in controlling body weight and composition. Therefore the identified SNP could affect FTO function or expression.

The rs9939609 was the most replicated SNP associated with obesity across the world, nevertheless, none of the study showed evidence of this SNP associated with overweight in the sample. This means that the FTO risk allele has a dominant effect on individuals with higher BMI; hence the association was detected in severe obesity rather than in overweight population [26].

There are 56 racial groups in China, Han Chinese constitutes more than 90% of China's population and is the largest ethnic group in the world, making up 20% of the entire global human population. The present study is to test whether common FTO gene SNPs are associated with obesity or related anthropometric traits in adolescents of Han Chinese. So far the data about the association of FTO with obesity in adolescents of southern Han Chinese was limited [27]–[29], the samples of our study were randomly selected from Wuxi (southern city of China). So our study may enrich the information about the association of FTO with obesity in adolescents of southern Han Chinese.

In our results, the allele A frequency of rs9939609 was 9.5%, which was a little lower than in Chinese Taiwan populations (12.6%) [11]. But the A frequency in Chinese populations was significantly lower than that in European populations (45%) and African American (21%) [17], [20], these results showed there are significant ethnic differences in FTO gene SNP frequency.

In our study, we found that compared to control group, overweight group has higher AA genotype frequency. Similarly, the allele A was more frequent in overweight group than that in control group, but the difference was not statistically significant. These results were similar to the results obtained by Liu et al [26], and suggested that stronger statistically significant results might be obtained by increasing the sample size.

The difference in BMI between the rs9939609 TA+AA genotypes and wild TT genotypes is worth mentioning. BMI of subjects with A allele is higher than that with T allele (*p* = 0.038), though results failed to reach statistical significance on adjusting for multiple testing, which suggests that A allele of FTO rs9939609 might be associated with BMI in Han Chinese adolescents. It is also possible that the rs9939609 polymorphism does not play any direct functional role in the development of obesity, but it might be in linkage disequilibrium with other polymorphisms, which could account for our observations. Finally, one limitation of the present study is that because of the small sample size, there is a possibility of sampling biases.

In conclusion, our study has conducted a genetic association of the FTO polymorphisms with BMI, serum fasting plasm glucose (FPG), fasting insulin (FIns), triglycerid (TG) and cholesterol (TC).Our study found BMI of subjects with A allele of FTO rs9939609 is higher than that with T allele. Further studies on other polymorphisms from FTO and increasing the sample size are needed, to establish the genetic basis contributing to the risk of obesity in Chinese population.
